# Epstein-Barr viral microRNAs target caspase 3

**DOI:** 10.1186/s12985-016-0602-7

**Published:** 2016-08-26

**Authors:** Cecelia Harold, Diana Cox, Kasandra J. Riley

**Affiliations:** 1 Department of Chemistry, Rollins College, Winter Park, FL 32789 USA; 2 Present Address: Albert Einstein College of Medicine, Bronx, NY 10461 USA; 3 Present Address: Baylor College of Medicine, Houston, TX 77030 USA

**Keywords:** Epstein-Barr virus, microRNA, Caspase 3, Burkitt’s lymphoma

## Abstract

The Epstein-Barr virus (EBV) is a ubiquitous herpesvirus that transforms B cells and causes several malignancies including Burkitt’s lymphoma. EBV differentially expresses at least 49 mature microRNAs (miRNAs) during latency in various infected epithelial and B cells. Recent high-throughput studies and functional assays have begun to reveal the function of the EBV miRNAs suggesting roles in latency, cell cycle control, and apoptosis. In particular, the central executioner of apoptosis, Caspase 3 (CASP3), was proposed as a target of select EBV miRNAs. However, whether CASP3 is truly a target of EBV miRNAs, and if so, which specific miRNAs target CASP3 is still under debate. Based on previously published high-throughput biochemical data and a bioinformatic analysis of the entire CASP3 3′-UTR, we identified 12 EBV miRNAs that have one or more seed binding sites in the CASP3 3′-UTR. We individually tested all 12 miRNAs for repression of CASP3 in luciferase reporter assays, and nine showed statistically significant (*P* < 0.001) repression of a full-length CASP3 reporter. Further, three EBV miRNAs, including BART22, exhibited repression of endogenous CASP3 protein. These data confirm that CASP3 is a direct target of specific EBV BART miRNAs.

**Electronic supplementary material:**

The online version of this article (doi:10.1186/s12985-016-0602-7) contains supplementary material, which is available to authorized users.

## Background

Epstein-Barr virus (EBV; human herpesvirus 4) persists in more than 95 % of the adult human population. Initial EBV infection is either asymptomatic or manifests as self-limiting mononucleosis. However, in a subset of cases, EBV is also associated with the oncogenic transformation of cells, resulting in malignancies including Burkitt’s lymphoma (BL) and several epithelial cell cancers (reviewed in [1]). After more than 50 years of study, the role of EBV in cancer is still under investigation. In addition to ~80 proteins and two ~170 nucleotide (nt) noncoding RNAs, EBV differentially expresses at least 49 mature microRNAs (miRNAs) from 25 precursors [2–4]. MiRNAs are ~22 nt noncoding RNAs that direct the modest downregulation of specific human and viral transcripts in diverse multicellular organisms. EBV miRNA expression varies widely in different cell types, latency stages, and strains of the virus [5, 6]. Select human and EBV miRNAs are thought to contribute to the cancer phenotype in both lymphoid and epithelial tumor types and are therefore considered possible therapeutic targets [7–12].

EBV precursor miRNAs arise from two genomic clusters: three surrounding the BHRF1 gene (BHRF1-1, 1-2, and 1-3), and 22 within the BART transcripts (BamHI A rightward transcripts; BARTs1-22) [2]. The BARTs are numbered in order of discovery and not in order of their appearance in the EBV genome [2–4]. Most human and viral miRNAs undergo a standard biogenesis pathway beginning with RNA polymerase II transcription of the primary-miRNA. In two steps, the first in the nucleus and the second in the cytoplasm, RNAse III enzymes sequentially process the primary-miRNA into the intermediate precursor-miRNA hairpin and final mature miRNA. One of the two strands is bound to core protein Argonaute and a target mRNA within the RNA-induced Silencing Complex (RISC). The single-stranded mature miRNA directs RISC to specific mRNA targets, typically via limited base-pairing to the mRNA 3′-untranslated region (3′-UTR) [13]. Key to downregulation is the base pairing interaction between the mRNA and 6-8 nt of the miRNA. This “seed region,” defined minimally as base pairing including nts numbered 2-7 from the 5′ end of the miRNA ([14]; Table 1), is crucial to the contacts between the miRNA, mRNA, and Argonaute protein [15]. Different patterns of base pairing can yield different degrees of repression; multiple sites are additive or synergistic, depending on their orientation [16]. Because the function of a given miRNA is determined by the mRNAs it targets, we and others conducted global biochemical and bioinformatic analyses predicting interactions between EBV and human miRNAs and human transcripts [11, 17, 18]. These studies provided thousands of predictions for miRNA-mRNA interactions, but further biochemical experimentation is required to confirm bona fide interactions between EBV miRNAs and human transcripts.

**Table 1:**
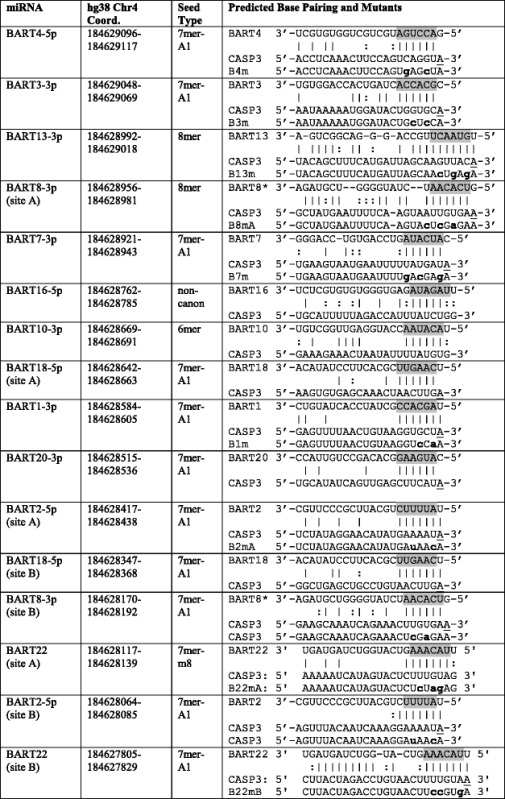
Tested interactions between EBV miRNAs and the full-length CASP3 3’UTR

Human Caspase 3 3’UTR (chr4:184627696-184629271; GRCh38/hg38 assembly) was searched for seed base pairing (nt 2-7 highlighted in gray, A1 position underlined), categorized by standard definitions [27]. “Non-canon” refers to the non-canonical site proposed by Veriede et al. [25]. All miRNA sequences are from MirBASE v. 21 [28]. Mutated sites in reporters are noted in bold lowercase letters

Three types of studies have demonstrated the important and complex role of EBV miRNAs in apoptosis. Phenotypic experiments implicated the BHRF1 miRNAs in apoptotic inhibition and promotion of the cell cycle during the early phase of infection of human primary B cells [12, 19]. Expression of clusters of BART miRNAs [20] or select BART miRNAs alone (BARTs 3, 6, 8, 16, and 22; [11]) is sufficient to exert an anti-apoptotic phenotype in AGS gastric carcinoma cells. Two different high-throughput studies of EBV miRNA targeting showed statistically significant enrichment of transcripts associated with apoptosis in B cells infected with different strains of EBV [17, 18]. In our published study of Jijoye cells [18], a BL cell line that expresses all known EBV miRNAs, 1664 human 3′-UTRs were identified as targets of the 12 most abundant EBV miRNAs, and approximately eight percent of these (132 transcripts) were associated in some way with apoptosis. Direct targets of EBV miRNAs were identified using standard reporter assays to validate base pairing between one or more EBV miRNAs and mRNA targets, including pro-apoptotic BBC3/PUMA [21], BCL2L11/BIM [20], BCL2L8/BAD [22], and anti-apoptotic BID [8]. Thus, the BART miRNAs directly downregulate both pro-and anti-apoptotic mediators that may vary by cell type.

Perhaps the most compelling mechanism for EBV miRNA inhibition of apoptosis is the potential repression of Caspase 3 (CASP3), the central proteolytic executioner of apoptosis [23], first identified as a potential direct EBV miRNA target in our high-throughput study [18]. Multiple groups have attempted to illuminate the role of EBV miRNAs in the regulation of CASP3. In an indirect assay for CASP3 regulation by EBV miRNAs, B cells infected by EBV lacking BART miRNAs expressed higher CASP3 and were more resistant to mitochondria-mediated apoptosis-inducing drugs relative to cells infected with intact EBV [24]. In a direct assay for miRNA repression, slight (~20 %) repression of a CASP3-luciferase reporter was observed after transfection of synthetic BARTs 1-3p and 16. Further, CASP3 protein levels decreased upon ectopic BART expression and increased upon depletion of EBV from S1-1 BL cells [25]. However, significant repression of a CASP3-luciferase reporter was not observed for BARTs 3, 6, 8, 16, or 22, when each was expressed from a lentiviral vector transfected into HEK293T cells, in spite of seed sequence sites in the CASP3 3′-UTR for each of these miRNAs [11]. Differences in experimental approach have likely led to these inconsistent results. Regardless, other groups have found that CASP3 protein is consistently lower in BL and epithelial cells expressing clusters of the BART miRNAs relative to cells without BARTs [20, 24, 25].

An individual study of each EBV miRNA with a potential binding site in CASP3 has not yet been undertaken, so it is unclear if single EBV miRNAs directly target CASP3. If single miRNAs are used as therapeutic targets, it is important to identify the unique targets/function of each. We employed systematic testing with standard reporter assays and Western blots to address our initial observation that CASP3 is a target of multiple EBV BART miRNAs [18] and clarify the role of EBV miRNAs in the regulation of CASP3. Nine EBV miRNAs bind to 13 specific locations on the CASP3 3′-UTR; three of the nine miRNAs detectably repress endogenous CASP3 protein independently. This is a critical step in detailing the mechanism by which EBV regulates apoptosis and could provide clues for future rational drug design.

## Methods

### Bioinformatic analyses

**Fig. 1:**
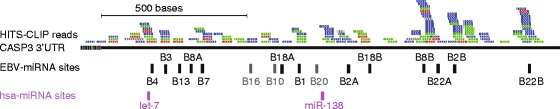
Caspase 3 has 16 predicted sites for EBV miRNA base pairing, 15 of which align with HITS-CLIP data. HITS-CLIP high-throughput sequencing reads (0 or 1 mismatch; ≥25 nt long) from six biological replicates of Ago-bound RNAs in Jijoye BL cells (unique reads, one color per biological replicate) mapped to scale on the Caspase 3 3′-UTR (1576 nt; RefSeq ID: NM_004346.3). Predicted sites of miRNA seed sequence binding for EBV miRNAs (black) and human miRNAs (purple) are noted below, with non-verifiable EBV miRNA sites in gray. When two sites for a given miRNA are present, they are labeled “A” and “B” from 5′ to 3′, respectively. Sites for BARTs 4, 7, and 22B met our initial HITS-CLIP reproducibility thresholds (three or more experimental replicates, peak height of five or more; [18])

Our previously published crosslinking and immunoprecipitation/high-throughput sequencing (HITS-CLIP) experiment identified CASP3 as a putative target of one or more EBV miRNAs, but we had not examined the CASP3 3′-UTR in single nucleotide resolution [18]. High-confidence HITS-CLIP sequence fragments were mapped onto the human genome (hg 18) as in [18], and the UCSC Genome Browser (https://genome.ucsc.edu) was used to generate an image from our BED file to overlay with the 3′-UTR of CASP3 (Fig. 1). The CASP3 3′-UTR was searched by hand for the reverse complement of canonical seed binding sites (7mer-A1, 7mer-m8, and 8mer) for all 49 known EBV miRNAs, and genomic coordinates of potential EBV miRNA binding sites were confirmed using the BLAST-Like Alignment Tool (BLAT) on the UCSC Genome Browser (Fig. 1, Table 1). All miRNA-mRNA pairs that met the minimal energetic requirements for base pairing were visualized using RNA22 [26]. Sites of potential human miRNA seed binding were identified by TargetScanHuman 7.0 [27].

### Constructs

The full length 3′-UTR of CASP3 (hg18, Chr4:185785817-185787419) was PCR amplified from genomic DNA and inserted downstream of firefly luciferase between NheI and XhoI in the pmiRGLO dual luciferase vector (Promega; Additional file 1: Table S1). Mutants were generated by site-directed mutagenesis (Stratagene) at the positions indicated in Table 1 with primers provided in Additional file 1: Table S1. All plasmids were verified by sequencing (Eurofins Genomics). Synthetic miRNAs (IDT) were synthesized according to the mature miRNA sequences in miRBase 21 [28] with a 5′-phosphate on the functional strand only, a mismatch at nt 2, and 2-nt 3′ overhangs, as in previous studies [14, 18, 29]. Pairs were annealed and confirmed on 3 % agarose gels according to [30]. The control miRNA “CTL” (5′-CTAGTATGACTAGTATGATCCGG top strand) has been used in previous studies [18]. CTL is a scrambled sequence with the same proportion of each nucleotide as hsa-miR-17, and it has no known biological function, seed sequence similarity to a known viral/human miRNA, or canonical targeting of CASP3.

### Luciferase assays

HEK293T cells (ATCC) in 24-well plates were co-transfected with 40 nM synthetic miRNA duplex and 2.5 ng pmiRGLO reporter plasmid using Lipofectamine 2000 (Invitrogen) according to the manufacturer’s instructions. Cells were lysed 24 h post-transfection, and the Dual-Luciferase Assay Reporter System (Promega) manufacturer’s protocol was performed with the GloMax Junior Luminometer (Promega). Firefly luciferase activity was normalized to the control Renilla luciferase activity in each well, and this ratio was then normalized to the control construct appropriate for each experiment. At least four independent transfections for each condition were measured. Excel was used to calculate the standard deviation for each data point, and two-tailed Student’s *t*-tests were used to compare samples as noted.

### Western blots

HEK293T cells were transfected in triplicate with the denoted synthetic miRNAs (40 nM) in the figures using Lipofectamine 2000 (Invitrogen). Protein lysates were prepared from cells, and Western blot analyses were performed as described (Wade and Allday, 2000) using NuPAGE 4-12 % gels and associated buffers (Invitrogen). Primary antibodies were anti-Caspase 3 (8G10; #9665) and anti-alpha-tubulin #2125 (Cell Signaling Technology). Western blots were quantified using ImageQuant software, with CASP3 levels normalized to the tubulin loading controls and these values normalized to the control transfection condition.

## Results

Argonaute HITS-CLIP experiments yield genome-wide maps of likely Argonaute and miRNA binding. Our previous HITS-CLIP data revealed potential interactions between EBV miRNAs and 132 host apoptotic mRNAs, including CASP3 [18]. We examined CASP3 because of its central role in apoptosis and the high reproducibility of the HITS-CLIP sequencing tags—sites of Argonaute binding—that overlapped with predicted seed binding sites for BARTs 4, 7, and 22 (Fig. 1). Because our HITS-CLIP experiment was performed in BL cells expressing all EBV miRNAs to varying degrees, we conducted a bioinformatic search for binding sites for all 49 EBV miRNAs and uncovered six more EBV miRNAs with one or more binding sites as well as a possible site for BART20 that did not overlap with our HITS-CLIP Argonaute binding sites (Fig. 1). In total, there are 14 putative miRNA binding sites for nine EBV miRNAs on the CASP3 3′-UTR. We also included two non-canonical sites for comparison: the previously published BART16 [25], which has a wobble base pair in the seed, and BART10, which has a 6mer site predicted in CASP3 (Fig. 1, Table 1).

**Fig. 2:**
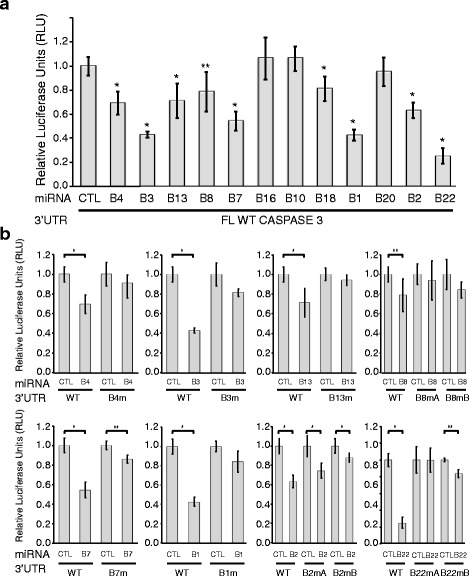
EBV miRNAs repress a full-length luciferase-Caspase 3 3′-UTR reporter. HEK293T cells were co-transfected with the designated luciferase-Caspase 3 reporters and either total synthetic control miRNA duplex (CTL) or an EBV miRNA predicted to base pair with the Caspase 3′-UTR (Table 1 includes all WT and mutant sequences). Firefly/Renilla luciferase ratios were normalized to the same reporter transfected with the negative control miRNA (CTL). **a** Wild type (WT) Caspase 3 is repressed significantly by nine EBV miRNAs. **b** WT repression is compared to loss of repression of the relevant mutant for each of the miRNAs that showed the greatest WT repression. In all luciferase assays, mean values were from at least four independent transfections. Error bars, standard deviation; *P* values from two-tailed Student’s *t*-tests of noted pairs, *P < 0.0001, **P < 0.01

In order to validate the bioinformatic and HITS-CLIP predicted interactions, the full-length 3′-UTR of CASP3 was cloned into a dual-luciferase reporter vector and co-transfected into HEK293T cells with individual synthetic miRNAs. Significant repression of the full-length CASP3 3′-UTR reporter occurred after transfection of synthetic BART 4, 3, 13, 8, 7, 18, 1, 2, or 22 (Fig. 2a). BART22 incited the greatest repression, and BARTs 16, 10, and 20 did not detectably repress CASP3.

The specificity of downregulation by CASP3 at the each of the most statistically significant and repressed (>20 %) binding sites was further confirmed by comparing wild-type 3′-UTR repression to the repression of a corresponding reporter with 2-3 point mutations in each seed binding region (Fig. 2b, Table 1). Three of the miRNAs that showed significant repression each had two possible sites (Fig. 1), so we individually mutated each site. The HITS-CLIP data show far less Argonaute binding at the BART8A, BART2A, and BART22A sites relative to their partner B sites (Fig. 1). In the case of BART2, the “B” site appeared to be more functional than the A. Mutation of the BART 22 “A” site alone was sufficient to de-repress the reporter almost completely (Fig. 2b), but mutation of the “B” site also showed significant derepression, suggesting that these two sites may work synergistically even though they are 312 nt apart (Table 1), because loss of either single site eliminates repression. In contrast, the weak BART8 repression via two sites appears to be roughly equal and additive (Fig. 2b). In total, our luciferase assay validates direct, reproducible, varying repression of the CASP3 3′-UTR reporter by BARTs 4, 3, 13, 8, 7, 1, 2, and 22.

**Fig. 3:**
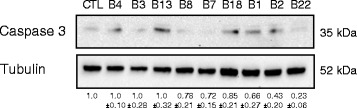
Select EBV miRNAs repress endogenous Caspase 3 protein. HEK293T cells were transfected with the denoted control or EBV miRNA. Western blots of extracts prepared 24 h post-transfection were probed for endogenous proteins with anti-Caspase 3 or anti-tubulin antibodies. Normalized CASP3 levels from triplicate experiments are reported below with the S.E.M

Given that these miRNAs significantly repress protein made from a reporter construct, we hypothesized that we might also see an effect on endogenous levels of CASP3 protein. Thus, we transfected individual synthetic EBV miRNAs into HEK293T cells, which lack endogenous EBV miRNAs, to confirm downregulation of endogenous CASP3 protein by Western blotting. CASP3 was downregulated most significantly by BART22 but also weakly by BARTs 8, 7, 1, and 2 (Fig. 3).

## Discussion

In the time since the publication of our high-throughput experiment that proposed CASP3 as a target of EBV miRNAs, several groups have published conflicting evidence of this interaction [11, 18, 22, 24, 25]. Here we confirmed that human CASP3 is a direct target of nine EBV BART miRNAs in luciferase assays by testing all canonical, predicted sites. Three of these miRNAs detectably, independently repressed endogenous CASP3 in HEK293T cells.

To complement our reporter assays, we used high levels of individual synthetic EBV miRNAs to demonstrate detectable repression of endogenous CASP3 protein on the part of select EBV miRNAs (Fig. 3). The levels of miRNA used in this proof-of-principle experiment are probably not physiologically attainable, but others have documented the effect of EBV miRNA expression on CASP3 protein levels and downstream effects on its substrate PARP (a measure of CASP3 activity) in both B cells and epithelial cells that are models for EBV infection. For example, CASP3 and cleaved PARP increase when AGS epithelial cells are treated with an inhibitor of BART20-5p. While BART20-5p is not likely a direct repressor of the CASP3 transcript, it represses BAD, a pro-apoptotic protein upstream of CASP3 [22]. Similarly, in EBV-infected B cells, CASP3 protein increases when all of the BARTs are deleted from the M81 strain [24]. Because the BART miRNAs target so many apoptotic transcripts [11, 17, 18], the effect of each individual EBV miRNA on CASP3 protein levels may vary. Thus, the luciferase assay is perhaps the best way to probe direct targeting of a given transcript.

The dual luciferase reporter assay undertaken in a cell line lacking relevant endogenous miRNAs remains the gold standard in the validation of direct targeting by miRNAs. Kang et al. (2015) recently conducted a reporter assay experiment similar to ours but only tested five miRNAs that had exerted an anti-apoptotic phenotype when expressed alone in their epithelial (gastric carcinoma) cell model (BARTs 3, 6, 8, 16, 22). The authors concluded that none of these miRNAs significantly repressed their CASP3 luciferase reporter in HEK293T cells. This conclusion may be explained by two key differences in our experimental designs: (1) the differing identity of our negative controls, and (2) the origin of our miRNAs. Cognizant that ectopic expression/transfection of both miRNAs and luciferase reporters skews the natural stoichiometry within a cell [31], and synthetic miRNAs accumulate well above physiologically attainable levels [32], all of our data are normalized to repression by a equal amounts of a transfected synthetic miRNA that does not repress our target of interest rather than a “mock” or “empty vector” transfection. Our synthetic miRNA duplexes may have accumulated to higher levels relative to the reporter than the miRNAs expressed from transduced lentiviral vectors employed by Kang et al, since those miRNAs presumably required standard biogenesis processing.

The BART miRNAs are expressed together in large clusters and likely operate in tandem [3, 19, 33]. Multiple miRNA binding sites in a single 3′-UTR are additive, so major targets of EBV miRNAs would have several miRNA binding sites, as appears to be the case for CASP3. Indeed, expression of BART miRNA clusters in B cells and AGS epithelial cells correlates with CASP3 depletion [20, 24]. If miRNA seed binding sites are 8 ~ 40 nt separated, repression is likely synergistic [14]. In CASP3 we noted one such potential case: the seed site for BART4 is 10 nt away from the seed site for human miRNA let-7. Further, while not highly expressed, let-7 is present in EBV+ Jijoye cells at approximately the same level as EBV-miR-BART5 or BART20-3p [18]. Human and viral miRNAs have been previously shown to co-target transcripts [17, 18], and it is possible that the effectiveness of BART4 repression is enhanced by its proximity to the let-7 site. TargetScan Human identified one other conserved but weaker potential binding site for a human miRNA: hsa-miR-138 (Fig. 1) [27]. MiR-138 is expressed at levels comparable to BART5 and let-7 in Jijoye cells [18], but our HITS-CLIP data do not overlap with this site, and no others have validated its direct regulation of CASP3. Consistent with this is the recent finding that miR-138 inhibits TNF-α-induced apoptosis in the human intervertebral disc, where an inhibitor of miR-138 dramatically suppressed the expression of cleaved CASP3 [34].

A previous study showed downregulation of CASP3 by BARTs 1-3p and 16 [25]. While our luciferase assay showed CASP3 repression upon treatment with BART1, we, like others [11], were unable to confirm their findings in the case of BART16. This is probably explained by the absence of true seed binding sites for BART16. The potential binding sites on the 3′-UTR of CASP3 contain a non-canonical Watson-Crick base pair in one nucleotide position of the seed region (Table 1). Non-canonical binding has been reported between human miRNAs and mRNAs, including seed regions binding with a G:U wobble base; however, the repression by these sites are not as significant as that of canonical sites [35, 36]. Similarly, the weaker 6mer site for BART10 did not exhibit repression of CASP3. Interestingly, the bioinformatically predicted site for BART20 was not validated by either HITS-CLIP data nor luciferase assays in spite of a predicted 7mer-A1 binding site, underscoring the need to move beyond bioinformatic predictions into the realm of biochemical testing before drawing conclusions.

Kaposi’s sarcoma herpesvirus (KSHV), a human gamma-herpesvirus responsible for primary effusion lymphoma (PEL) and Kaposi’s sarcoma (KS), also expresses miRNAs that repress apoptotic targets including CASP3 [37, 38]. Though KSHV and EBV miRNA sequences are not conserved and show functional differences with respect to the latent-to-lytic switch, targeting the same apoptotic transcript potentially shows a similar method for controlling apoptosis in the two viruses [39, 40].

A decrease in apoptotic activity of EBV-positive cells could promote malignancy in conjunction with the deactivation of other important cellular pathways by EBV miRNAs. The 100+ other apoptotic transcripts with putative EBV miRNA binding sites should be studied to elucidate the mechanism by which EBV controls host cell apoptosis and further our understanding of how the virus causes malignancy. While the EBV BART miRNAs work in tandem to repress CASP3, therapeutically targeting a large number of miRNAs is likely more challenging and may have more off-target consequences. Knowing that single miRNAs can repress a key target may help us better conduct future rational drug design regarding EBV and BL.

## Additional file

Additional file 1: Table S1. Oligonucleotides used in this study. (DOC 60 kb)

## Abbreviations

EBV: Epstein-Barr virus
CASP3: Caspase 3
nt: nucleotide
3′-UTR: 3′-untranslated region
BL: Burkitt’s lymphoma
RISC: RNA-induced silencing complex
miRNA(s): microRNA(s)
BART: BamHI A rightward transcripts

## References

[1] Houldcroft CJ, Kellam P (2015). Host genetics of Epstein-Barr virus infection, latency and disease. Rev Med Virol.

[2] Pfeffer S, Zavolan M, Grässer FA, Chien M, Russo JJ, Ju J, John B, Enright AJ, Marks D, Sander C, Tuschl T (2004). Identification of virus-encoded microRNAs. Science.

[3] Cai X, Schäfer A, Lu S, Bilello JP, Desrosiers RC, Edwards R, Raab-Traub N, Cullen BR (2006). Epstein-Barr virus microRNAs are evolutionarily conserved and differentially expressed. PLoS Pathog.

[4] Grundhoff A, Sullivan CS (2011). Virus-encoded microRNAs. Virology.

[5] Nourse JP, Crooks P, Keane C, Nguyen-Van D, Mujaj S, Ross N, Jones K, Vari F, Han E, Trappe R, Fink S, Gandhi MK (2012). Expression profiling of Epstein-Barr virus-encoded microRNAs from paraffin-embedded formalin-fixed primary Epstein-Barr virus-positive B-cell lymphoma samples. J Virol Methods.

[6] Yang H-J, Huang T-J, Yang C-F, Peng L-XL-X, Liu R-Y, Yang G-D, Chu Q-Q, Huang J-L, Liu N, Huang H-B, Zhu Z-Y, Qian C-N, Huang B-J (2013). Comprehensive profiling of Epstein-Barr virus-encoded miRNA species associated with specific latency types in tumor cells. Virol J.

[7] Shin VY, Chu K-M (2014). MiRNA as potential biomarkers and therapeutic targets for gastric cancer. World J Gastroenterol.

[8] Shinozaki-Ushiku A, Kunita A, Isogai M, Hibiya T, Ushiku T, Takada K, Fukayama M (2015). Profiling of virus-Encoded MicroRNAs in Epstein-Barr virus-associated gastric carcinoma and their roles in gastric carcinogenesis. J Virol.

[9] Linnstaedt SD, Gottwein E, Skalsky RL, Luftig M a, Cullen BR (2010). Virally induced cellular microRNA miR-155 plays a key role in B-cell immortalization by Epstein-Barr virus. J Virol.

[10] Qiu J, Smith P, Leahy L, Thorley-Lawson DA (2015). The Epstein-Barr Virus Encoded BART miRNAs potentiate tumor growth In Vivo. PLOS Pathog.

[11] Kang D, Skalsky RL, Cullen BR (2015). EBV BART MicroRNAs target multiple pro-apoptotic cellular genes to promote epithelial cell survival. PLOS Pathog.

[12] Seto E, Moosmann A, Grömminger S, Walz N, Grundhoff A, Hammerschmidt W (2010). Micro RNAs of Epstein-Barr virus promote cell cycle progression and prevent apoptosis of primary human B cells. PLoS Pathog.

[13] Bartel DP. MicroRNAs: genomics, biogenesis, mechanism, and function. Cell. 2004;281–297. 10.1016/s0092-8674(04)00045-5 14744438

[14] Grimson A, Farh KK-H, Johnston WK, Garrett-Engele P, Lim LP, Bartel DP (2007). MicroRNA targeting specificity in mammals: determinants beyond seed pairing. Mol Cell.

[15] Schirle NT, Sheu-Gruttadauria J, MacRae IJ (2014). Structural basis for microRNA targeting. Science.

[16] Bartel DP (2009). MicroRNAs: target recognition and regulatory functions. Cell.

[17] Skalsky RL, Corcoran DL, Gottwein E, Frank CL, Kang D, Hafner M, Nusbaum JD, Feederle R, Delecluse H-J, Luftig MA, Tuschl T, Ohler U, Cullen BR (2012). The Viral and Cellular MicroRNA Targetome in lymphoblastoid cell lines. PLoS Pathog.

[18] Riley KJ, Rabinowitz GS, Yario TA, Luna JM, Darnell RB, Steitz JA (2012). EBV and human microRNAs co-target oncogenic and apoptotic viral and human genes during latency. EMBO J.

[19] Bernhardt K, Haar J, Tsai M-H, Poirey R, Feederle R, Delecluse H-J (2016). A Viral microRNA cluster regulates the expression of PTEN, p27 and of a bcl-2 Homolog. PLoS Pathog.

[20] Marquitz AR, Mathur A, Nam CS, Raab-Traub N (2011). The Epstein-Barr virus BART microRNAs target the pro-apoptotic protein Bim. Virology.

[21] Choy EY-W, Siu K-L, Kok K-H, Lung RW-M, Tsang CM, To K-F, Kwong DL-W, Tsao SW, Jin D-Y (2008). An Epstein-Barr virus-encoded microRNA targets PUMA to promote host cell survival. J Exp Med.

[22] Kim H, Choi H, Lee SK (2015). Epstein-Barr Virus MicroRNA miR-BART20-5p Suppresses Lytic induction by inhibiting BAD-Mediated caspase-3-dependent apoptosis. J Virol.

[23] Green DR, Llambi F (2015). Cell death signaling. Cold Spring Harb Perspect Biol.

[24] Lin X, Tsai M-H, Shumilov A, Poirey R, Bannert H, Middeldorp JM, Feederle R, Delecluse H-J (2015). The Epstein-Barr Virus BART miRNA cluster of the M81 strain modulates multiple functions in primary B cells. PLoS Pathog.

[25] Vereide DT, Seto E, Chiu Y-F, Hayes M, Tagawa T, Grundhoff A, Hammerschmidt W, Sugden B (2014). Epstein-Barr virus maintains lymphomas via its miRNAs. Oncogene.

[26] Miranda KC, Huynh T, Tay Y, Ang Y-S, Tam W-L, Thomson AM, Lim B, Rigoutsos I (2006). A Pattern-based Method for the Identification of MicroRNA binding sites and their corresponding Heteroduplexes. Cell.

[27] Agarwal V, Bell GW, Nam J-W, Bartel DP. Predicting effective microRNA target sites in mammalian mRNAs. Elife. 2015;4. 10.7554/eLife.05005 PMC4532895 26267216

[28] Kozomara A, Griffiths-Jones S (2014). miRBase: annotating high confidence microRNAs using deep sequencing data. Nucleic Acids Res.

[29] Guo YE, Riley KJ, Iwasaki A, Steitz JA (2014). Alternative capture of Noncoding RNAs or protein-coding genes by Herpesviruses to alter host T cell function. Mol Cell.

[30] Tuschl T. Annealing siRNAs to produce siRNA duplexes. CSH Protoc. 2006;2006. 10.1101/pdb.prot4340 22485716

[31] Riley KJ, Steitz JA (2013). The “Observer Effect” in genome-wide surveys of protein-RNA interactions. Mol Cell.

[32] Riley KJ, Yario TA, Steitz JA (2012). Association of argonaute proteins and microRNAs can occur after cell lysis. RNA.

[33] Kanda T, Miyata M, Kano M, Kondo S, Yoshizaki T, Iizasa H (2015). Clustered microRNAs of the Epstein-Barr virus cooperatively downregulate an epithelial cell-specific metastasis suppressor. J Virol.

[34] Wang B, Wang D, Yan T, Yuan H (2016). MiR-138-5p promotes TNF-α-induced apoptosis in human intervertebral disc degeneration by targeting SIRT1 through PTEN/PI3K/Akt signaling. Exp Cell Res.

[35] Seok H, Ham J, Jang E-S, Chi SW (2016). MicroRNA target recognition: insights from Transcriptome-wide non-canonical interactions. Mol Cells.

[36] Loeb GB, Khan AA, Canner D, Hiatt JB, Shendure J, Darnell RB, Leslie CS, Rudensky AY (2012). Transcriptome-wide miR-155 binding map reveals widespread noncanonical microRNA targeting. Mol Cell.

[37] Haecker I, Gay LA, Yang Y, Hu J, Morse AM, McIntyre LM, Renne R (2012). Ago HITS-CLIP expands understanding of Kaposi’s sarcoma-associated herpesvirus miRNA function in primary effusion lymphomas. PLoS Pathog.

[38] Suffert G, Malterer G, Hausser J, Viiliäinen J, Fender A, Contrant M, Ivacevic T, Benes V, Gros F, Voinnet O, Zavolan M, Ojala PM, Haas JG, Pfeffer S (2011). Kaposi’s Sarcoma Herpesvirus microRNAs target Caspase 3 and regulate apoptosis. PLoS Pathog.

[39] Kincaid RP, Sullivan CS (2012). Virus-encoded microRNAs: an overview and a look to the future. PLoS Pathog.

[40] Boss IW, Plaisance KB, Renne R (2009). Role of virus-encoded microRNAs in herpesvirus biology. Trends Microbiol.

