# Supplementary material for: Epstein-Barr viral microRNAs target caspase 3
Source: Virol J. 2016 Aug 26;13(1):145. doi: 10.1186/s12985-016-0602-7 (PMC5002152; doi:10.1186/s12985-016-0602-7)
Supplement: Additional file 1: Table S1. — Oligonucleotides used in this study. (DOC 60 kb) [file 12985_2016_602_MOESM1_ESM.doc]

**Supplemental Table 1**: Oligonucleotides used in this study

| | **Oligonucleotide Sequence** | **Purpose** | | --- | --- | | gatcgctagcAGAAATGGTTGGTTGGTGGTTTTTTTTAG | PCR primer for CASP3 3'UTR | | gatcctcgagCAGCAACAAAAAACTCAAAGACATTGG | PCR primer for CASP3 3'UTR | | GAAGCTACCTCAAACTTCCAGTgAGcTAGTTGCAATTGAATTAAATTAGG | SDM primer for CASP3 B4 mutation | | CCTAATTTAATTCAATTGCAACTAgCTcACTGGAAGTTTGAGGTAGCTTC | SDM primer for CASP3 B4 mutation | | GAATAAATAAAAATGGATACTGcTcCAGTCATTATGAGAGGCAATGATTG | SDM primer for CASP3 B3 mutation | | CAATCATTGCCTCTCATAATGACTGgAgCAGTATCCATTTTTATTTATTC | SDM primer for CASP3 B3 mutation | | GTTAATTTACAGCTTTCATGATTAGCAAcTgAgAGTGATGCTGTGCTATGAATTTTCAAG | SDM primer for CASP3 B13 mutation | | CTTGAAAATTCATAGCACAGCATCACTcTcAgTTGCTAATCATGAAAGCTGTAAATTAAC | SDM primer for CASP3 B13 mutation | | AAACATTGAAGTAATGAATTTTGACGAGATTCCCCCCACTTAAGACTGTG | SDM primer for CASP3 B7 mutation | | CACAGTCTTAAGTGGGGGGAATCTCGTCAAAATTCATTACTTCAATGTTT | SDM primer for CASP3 B7 mutation | | GGAGTTTTAACTGTAAGGTcCaACAATGCCCCTGGATCTAC | SDM primer for CASP3 B1 mutation | | GTAGATCCAGGGGCATTGTtGgACCTTACAGTTAAAACTCC | SDM primer for CASP3 B1 mutation | | GTGAATAAATTCTATAGGAACATATGAtAAcACAACTTAAATAATAAACAGTGGAATATA | SDM primer for CASP3 B2A mutation | | TATATTCCACTGTTTATTATTTAAGTTGTgTTaTCATATGTTCCTATAGAATTTATTCAC | SDM primer for CASP3 B2A mutation | | CTGTTGAAGTTTACAATCAAAGGAtAAcAGTAATGTTTTATACTGTTTACTGAAAG | SDM primer for CASP3 B2B mutation | | CTTTCAGTAAACAGTATAAAACATTACTgTTaTCCTTTGATTGTAAACTTCAACAG | SDM primer for CASP3 B2B mutation | | TTTTCTTACTAGACCTGTAACTTCCGTGAATACACATAGCATGTAATGGT | SDM primer for CASP3 B22B mutation | | ACCATTACATGCTATGTGTATTCACGGAAGTTACAGGTCTAGTAAGAAAA | SDM primer for CASP3 B22B mutation | |  |  | | P-UAGCACCGCUAUCCACUAUGUC | Top strand-synthetic BART1-3p (B1) | | CAUAGUGGAUAGCGGUGCCAUG | Bottom strand-synthetic BART1-3p (B1) | | P-UAUUUUCUGCAUUCGCCCUUGC | Top strand-synthetic BART2-5p (B2) | | AAGGGCGAATGCAGAAAACAAG | Bottom strand-synthetic BART2-5p (B2) | | P-CGCACCACUAGUCACCAGGUGU | Top strand-synthetic BART3-3p (B3) | | ACCUGGUGACUAGUGGUGAGUG | Bottom strand-synthetic BART3-3p (B3) | | P-GACCUGAUGCUGCUGGUGUGCU | Top strand-synthetic BART4 (B4) | | CACACCAGCAGCAUCAGGCCAC | Bottom strand-synthetic BART4 (B4) | | P-CAUCAUAGUCCAGUGUCCAGGG | Top strand-synthetic BART7-3p (B7) | | CUGGACACUGGACUAUGACGAC | Bottom strand-synthetic BART7-3p (B7) | | P-GUCACAAUCUAUGGGGUCGUAGA | Top strand-synthetic BART8-3p (B8) | | UACGACCCCAUAGAUUGUGUCAG | Bottom strand-synthetic BART8-3p (B8) | | P-UACAUAACCAUGGAGUUGGCUGU | Top strand-synthetic BART10-3p (B10) | | AGCCAACUCCAUGGUUAUGCACA | Bottom strand-synthetic BART10-3p (B10) | | P-UGUAACUUGCCAGGGACGGCUGA | Top strand-synthetic BART13-3p (B13) | | AGCCGUCCCUGGCAAGUUAGAGA | Bottom strand-synthetic BART13-3p (B13) | | P-UUAGAUAGAGUGGGUGUGUGCUCU | Top strand-synthetic BART16 (B16) | | AGCACACACCCACUCUAUCUUAAU | Bottom strand-synthetic BART16 (B16) | | P-UCAAGUUCGCACUUCCUAUACA | Top strand-synthetic BART18-5p (B18) | | UAUAGGAAGUGCGAACUUCAAC | Bottom strand-synthetic BART18-5p (B18) | | P-CAUGAAGGCACAGCCUGUUACC | Top strand-synthetic BART20-3p (B20) | | UAACAGGCUGUGCCUUCACGCC | Bottom strand-synthetic BART20-3p (B20) | | P-UUACAAAGUCAUGGUCUAGUAGU | Top strand-synthetic BART22 (B22) | | UACUAGACCAUGACUUUGUUACC | Bottom strand-synthetic BART22 (B22) | | P-CUAGUAUGACUAGUAUGAUCCGG | Top strand-synthetic control miRNA (CTL) | | GGAUCAUACUAGUCAUACUUGAC | Bottom strand-synthetic control miRNA (CTL) | |
| --- | --- | --- | --- | --- | --- | --- | --- | --- | --- | --- | --- | --- | --- | --- | --- | --- | --- | --- | --- | --- | --- | --- | --- | --- | --- | --- | --- | --- | --- | --- | --- | --- | --- | --- | --- | --- | --- | --- | --- | --- | --- | --- | --- | --- | --- | --- | --- | --- | --- | --- | --- | --- | --- | --- | --- | --- | --- | --- | --- | --- | --- | --- | --- | --- | --- | --- | --- | --- | --- | --- | --- | --- | --- | --- | --- | --- | --- | --- | --- | --- | --- | --- | --- | --- | --- | --- | --- | --- | --- | --- | --- | --- |

All oligonucleotides are written 5’ to 3’. Polymerase chain reaction (PCR) and site-directed mutagenesis (SDM) primer were synthesized without added phosphate groups. Top strands for synthetic miRNAs were phosphorylated (P) on the 5’ end.
